# Comprehensive genomic characterization of five canine lymphoid tumor cell lines

**DOI:** 10.1186/s12917-016-0836-z

**Published:** 2016-09-17

**Authors:** Sarah C. Roode, Daniel Rotroff, Kristy L. Richards, Peter Moore, Alison Motsinger-Reif, Yasuhiko Okamura, Takuya Mizuno, Hajime Tsujimoto, Steven E. Suter, Matthew Breen

**Affiliations:** 1 Department of Molecular Biomedical Sciences, College of Veterinary Medicine, North Carolina State University, CVM Research Building - Room 348, 1060 William Moore Drive, Raleigh, 27607 NC USA; 2 Bioinformatics Research Center, Department of Statistics, North Carolina State University, Raleigh, NC USA; 3 Department of Medicine, University of North Carolina, Chapel Hill, NC USA; 4 Comparative Medicine Institute, North Carolina State University, Raleigh, NC USA; 5 Cancer Genetics Program, Lineberger Comprehensive Cancer Center, University of North Carolina, Chapel Hill, NC USA; 6 Department of Pathology, Microbiology, and Immunology, College of Veterinary Medicine, University of California, Davis, CA USA; 7 Veterinary Teaching Hospital, Faculty of Agriculture, Iwate University, Morioka, Japan; 8 Laboratory of Veterinary Internal Medicine, Faculty of Agriculture, Yamaguchi University, Yamaguchi, Japan; 9 Graduate School of Agricultural and Life Sciences, University of Tokyo, Bunkyo, Japan; 10 Department of Clinical Sciences, College of Veterinary Medicine, North Carolina State University, CVM Research Building - Room 308, 1051 William Moore Drive, Raleigh, NC 27607 USA; 11 KLR current address: Department of Biomedical Sciences, Cornell University, Ithaca, NY USA

## Abstract

**Background:**

Leukemia/lymphoma cell lines have been critical in the investigation of the pathogenesis and therapy of hematological malignancies. While human LL cell lines have generally been found to recapitulate the primary tumors from which they were derived, appropriate characterization including cytogenetic and transcriptional assessment is crucial for assessing their clinical predictive value.

**Results:**

In the following study, five canine LL cell lines, CLBL-1, Ema, TL-1 (Nody-1), UL-1, and 3132, were characterized using extensive immunophenotyping, karyotypic analysis, oligonucleotide array comparative genomic hybridization (oaCGH), and gene expression profiling. Genome-wide DNA copy number data from the cell lines were also directly compared with 299 primary canine round cell tumors to determine whether the cell lines represent primary tumors, and, if so, what subtype each most closely resembled.

**Conclusions:**

Based on integrated analyses, CLBL-1 was classified as B-cell lymphoma, Ema and TL-1 as T-cell lymphoma, and UL-1 as T-cell acute lymphoblastic leukemia. 3132, originally classified as a B-cell lymphoma, was reclassified as a histiocytic sarcoma based on characteristic cytogenomic properties. In combination, these data begin to elucidate the clinical predictive value of these cell lines which will enhance the appropriate selection of in vitro models for future studies of canine hematological malignancies.

**Electronic supplementary material:**

The online version of this article (doi:10.1186/s12917-016-0836-z) contains supplementary material, which is available to authorized users.

## Background

Hematological diseases in humans are widely heterogeneous including numerous molecular subtypes with wide ranging prognoses and therapeutic responses. Oncogenesis is well understood for some subtypes, while the molecular changes leading to other subtypes remain unknown [1]. A similar level of molecular heterogeneity likely exists in the >1000 leukemia/lymphoma (LL) cell lines that have been described to date [2].

Although the use of cell lines provides numerous advantages, including ease of handling and manipulation, high homogeneity, provision of a continuous source of sample material, and accessibility to the scientific community [3], detailed characterization is crucial before they are used as an in vitro preclinical cancer models. Nearly 100 % of examined human LL cell lines carry stable genetic alterations and karyotypic changes that maintain the major features of the original cells [2] while a number of other human LL cell line studies have provided evidence that these cell lines have a high clinical predictive value that may translate into a favorable response rates in Phase II clinical trials [4, 5].

Cell line characterization minimally requires that a comprehensive set of immunophenotyping and cytogenetic data have been published [6]. However, with the recent explosion of advances in the genomics field, it is now possible to provide a more in-depth assessment of cytogenetic and transcriptional characteristics of cell lines that can provide further insight into biological processes including chromosomal translocations, signaling pathways, mutational analysis, gene dysregulation, and RNAi gene silencing [2]. Of the >1000 human LL cell lines described, ~40 % have been characterized in sufficient detail for accurate classification as discrete LL subtypes [6].

Spontaneously occurring lymphoid malignancies in dogs share the same histopathological and clinical features of their human couterparts, in addition to evolutionarily conserved chromosome aberrations and mutations, indicating shared pathogenesis across species [7–9]. There are only a small number of established canine LL cell lines, six of which have already been characterized at the genomic level [10, 11]. Similar to human LL cell lines, the importance of detailed genomic and phenotypic characterization in an effort to define a framework to assess their clinical predictive value was recently emphasized [12]. Five additional canine LL cell lines, CLBL-1, Ema, UL-1, TL-1 (Nody-1), and 3132, have been used previously in in vitro studies [13–21]. These cell lines have varying levels of characterization, none of which includes an in-depth genomic and transcriptomic approach [22–24].

We present a comprehensive characterization of five canine LL cell lines starting with an extended panel of immunophenotyping. High resolution oligonucleotide array comparative genomic hybridization (oaCGH) was performed to assess genome-wide copy number status, and multicolor fluorescence in situ hybridization (FISH) analysis was used to further identify copy number imbalances and structural changes in karyotype architecture. Transcription status of each cell line was investigated using high-density array based gene expression profiling (GEP) and quantitative reverse transcriptase polymerase chain reaction (qRT-PCR). Additionally, genome wide copy number data of each cell line were compared with data from primary canine round cell tumors to further confirm their classification and relevance as in vitro preclinical models of lymphoid neoplasia for canine and comparative medicine.

## Methods

### Canine LL cell lines

Five previously established canine LL cell lines with varying levels of initial characterization were included in this study: CLBL-1 [22] (kind gift from Dr. Barbara Rutgen, University of Veterinary Medicine Vienna, Austria, Ema [23] (kind gift from Dr. Takuya Mizuno, Yamaguchi University, Japan), TL-1 [23] (Nody-1, kind gift from Dr. Yasuhiko Okamura, Iwate University, Japan), UL-1 [23] (kind gift from Dr. Hajime Tsujimoto, University of Tokyo, Japan), and 3132 [24, 25] (kind gift from Dr. Mark Holmes, University of Cambridge, UK). All cell lines were maintained at 37 °C and 5 % CO_2_ in RPMI-1640 culture medium (Mediatech, Hendon, VA) supplemented with 10 % fetal bovine serum (FBS, Mediatech), 2 mM Glutamax (Life Technologies, Grand Island, NY), and 100 μg/ml Primocin (Invivogen, San Diego, CA) and tested negative using a PCR Mycoplasma test kit (Applichem, Cheshire, CT).

### Immunophentyping

Immunophenotyping of each line was completed using flow cytometry as previously described [11, 26] at the UC Davis Leukocyte Antigen Biology Laboratory using a panel of monoclonal antibodies reactive with canine leukocyte antigens, including CD1a, CD1c, CD3, CD4, CD5, CD8α, CD8β, CD11a, CD11b, CD11c, CD11d, CD14, CD18, CD21, CD34, CD45, CD45RA, CD49a, CD54, CD79α, CD80, CD86, DM5, MHC-II, 5G2, AG5, TCRαβ, TCRγδ, TCRαβ, TCRCCγδ, and Thy-1 (CD90). Analysis at the NCSU Clinical Immunology Laboratory was also performed using a smaller number of antibodies, including CD3, CD4, CD5, CD8α, CD21, CD34, and CD79α/β.

### PARR

Polymerase chain reaction for antigen receptor rearrangement (PARR) was completed as previously described [27, 28] to assess clonality and, possibly lineage. PCR products were separated using capillary gel electrophoresis (QIAxcel Electrophoresis System, Qiagen, Valencia, CA). A clonal sample was determined if one or more discrete bands were seen on the gel, and a polyclonal sample was determined if multiple bands or a smear of amplicons were seen. A negative sample was determined if no bands were seen.

### Isolation of cell line DNA and RNA and generation of metaphase preparations

Aliquots of 1 × 10^7^cells were removed from the same culture flask at the same time point for isolation of DNA and RNA and preparation of metaphase chromosome preparations to ensure consistency in downstream analyses. DNA was isolated using the DNeasy Blood and Tissue kit (Qiagen, Valencia, CA) and manufacturer’s protocol, and quantity and quality of DNA were evaluated using spectrophotometry (260/280 > 1.8) and gel electrophoresis. RNA was isolated using the RNeasy Plus Mini Kit (Qiagen) and manufacturer’s protocol, and assessed using the 2100 Bioanalyzer RNA 6000 Nano Kit (Agilent Technologies, Santa Clara, CA) to confirm an RNA integrity number (RIN) >9.0. Metaphase chromosome preparations were prepared from each cell line as previously described [29] using conventional techniques of colcimid arrest (final concentration of 50 ng/ml for 1 h), hypotonic treatment, and methanol-glacial acetic acid fixation prior to being dropped onto glass slides.

### Identification of copy number aberrations (CNAs) using oaCGH

oaCGH was completed as previously described using a 180,000 feature canine oligonucleotide array (Agilent Technologies) with repeat-masked 60mer oligonucleotides spaced ~13 kb across the genome [30]. An equimolar pool of DNA from 25 clinically healthy female dogs was used as a common reference for all cell lines. Cell line and reference DNA was labeled with Cyanine 3-dUTP and Cyanine 5-dUTP, respectively, using the Agilent Enzymatic Labeling Kit, and probe hybridization, array washing, and scanning was performed as described elsewhere [30].

Scan data were processed using Feature Extraction v.10.10 software (Agilent Technologies) and imported into Nexus Copy Number v7.5 (Biodiscovery, Hawthorne, CA). Raw data were evaluated to identify and exclude probes displaying non-uniform hybridization or signal saturation, and copy number calls were made using Biodiscovery’s FASST2 segmentation algorithm. Copy number calls were based on a minimum of three consecutive probes per segment, and mean log2 cell line:reference thresholds of +/− 0.2 were used to define gain and loss, respectively. Genes within the defined intervals were identified using the UCSC canine genome browser (CanFam2 assembly; http://genome.ucsc.edu/) and the NCBI gene database (http://www.ncbi.nlm.nih.gov/gene). Genes previously associated with cancer were based on those reported in the Cancer Gene Census (http://cancer.sanger.ac.uk/cosmic/census) [31].

Further statistical analyses using the Feature Extraction data were performed using R [32]. The signals (rProcessedSignal and gProcessedSignal) were normalized using the following equations:

1 $$ a = \mathrm{l}\mathrm{o}{\mathrm{g}}_2\left( rprocessedSigna/ gProcessedSignal\right) $$

2 $$ ProcessedRatio=\left[a\hbox{--} mode\ (a)\right]/ MAD(a) $$

Where, *Processed ratio* is the centered and normalized ratio of the Agilent processed fluorescent signals. Segmentation was performed across all chromosomes using circular binary segmentation [33]. Data were further dichotomized as gain (1), no change (0), or loss (−1), based on segments that were +/− 3 MAD (mean absolute deviation) from the median of each sample’s response across all chromosomes. Hierarchical clustering of the five cell lines was performed using dichotomous data using Euclidean distance and Ward’s method. Additionally, hierarchical clustering of the cell lines with 299 canine primary round cell tumors including 123 leukemias [34], 106 lymphomas (Thomas et al., in preparation), and 70 histiocytic malignancies (Kennedy et al., in preparation) was performed using segmented data using Euclidean distance and Ward’s method. Tumor type and subtype were both annotated on the heatmap.

### FISH analysis

FISH of all cells lines was performed as previously described [29] to evaluate structural changes and verify oaCGH copy number data using panels of clones from the CHORI-82 dog bacterial artificial chromosome (BAC) library (www.chori.org). Initially, two clones (326 K03 and 330E21), previously determined to hybridize to the centromeric regions of canine autosomes [35], were fluorescently labeled and hybridized to metaphase preparations of each cell line. Centromeric signals were used to properly orient the chromosomes, aid in confirming modal chromosome count, and identify bi-armed chromosomes.

Twenty additional BAC clones were selected to contain known oncogenes and tumor suppressor genes that met two of the three following criteria: (1) located in a region of CNA in at least one of the five cells lines, (2) displayed differential expression between cell lines based on the GEP, and (3) have been associated with human and/or canine lymphoid malignancies in prior studies [8, 9, 36, 37, 38, 39, 40, 41, 42, 43, 44, 45, 46]. The BAC clones selected represented the following genes: *BCL11B, IGH, VEGFA, CCNC, FOXO3A, CDKN2A, MYC, KIT, CDK6, EZH2, MYCBP2, FLT3, PTEN, HEY1, E2F5, NFKB2, ERG, MLLT2, CD83,* and *DEK* (Table 1). The 20 clones were divided into four panels of five for multicolor FISH, and each panel was hybridized to metaphase spreads of each cell line and healthy dog controls. Copy number status of each probe was scored in at least 50 cells of each cell line and normal controls.

**Table 1:** BAC clones from CHORI-82 dog library selected to represent 20 cancer-related genes for FISH analysis. Chromosome locations based on the CanFam2 genome assembly are noted

Gene	BAC clone	Chromosome	Start (bp)	Stop (bp)
BCL11B	326-K01	8	70,661,395	70,752,579
IGH	027-N17	8	75,997,304	76,191,846
VEGFA	152-L05	12	15,212,673	15,228,610
CCNC	268-D08	12	60,739,913	60,764,849
FOXO3A	048-I05	12	68,583,078	68,701,688
CDKN2A	325-C12	11	44,255,629	44,256,009
MYC	335-M01	13	28,238,008	28,242,545
KIT	98-B16	13	50,017,518	50,212,194
CDK6	181-D14	14	21,147,772	21,367,160
EZH2	300-P18	16	4,905,169	4,971,032
MYCBP2	216-G13	22	33,561,172	33,820,510
FLT3	062-D23	25	14,581,755	14,658,045
PTEN	521-G14	26	40,921,802	40,981,821
HEY1	484-E08	29	30,184,049	30,186,972
E2F5	157-A19	29	34,748,851	34,758,529
NFKB2	001-D14	28	17,903,193	17,910,910
ERG	100-F17	31	35,578,420	35,760,306
MLLT2	468-E14	32	13,354,180	13,586,952
CD83	127-B24	35	16,354,899	16,533,175
DEK	517-A02	35	20,035,294	20,172,093

### GEP analysis

Total RNA from each cell line was used to perform gene expression profiling (GEP) as described elsewhere [11, 47] using the GeneChip Canine Genome 2.0 array (Affymetrix, Santa Clara, CA) which is comprised of 18,000 *Canis familiaris* mRNA transcripts and over 20,000 non-redundant predicted genes. Additionally, total RNA was isolated as described from lymph nodes harvested from six healthy mixed breed dogs that showed no evidence of lymphoid neoplasia at necropsy [11]. Microarrays were processed by the Lineberger Functional Genomic Core Facility at the University of North Carolina Chapel Hill. Total RNA (1 μg) was processed for microarray hybridization using the MessageAmp II-Biotin Enhanced Kit (Ambion, Life Technologies, Grand Island, NY) and hybridization was performed according to Affymetrix technical protocols. GEP analysis was performed using GeneSpring GX v12 (Agilent Technologies). Expression array data were normalized using the GC-RMA procedure [48] and signals were median-centered across all arrays. Data were filtered to remove probe sets with limited variation (standard deviation <2) across all arrays, and fold change analysis was performed for each cell line using the averaged expression data from the normal lymph node controls as baseline. Additionally, unsupervised hierarchical clustering analysis was performed across all cell lines and controls using the filtered probe set. Functional analysis was performed by evaluating for enrichment in genes that were up- or down-regulated in each cell line by >5-fold compared to normal lymph node controls. Enrichment analysis in Gene Ontology (GO) biological processes and Kyoto Encyclopedia of Genes and Genome (KEGG) Pathways was completed using the Database for Annotation, Visualization, and Integrated Discovery (DAVID) v 6.7 [49, 50].

### qRT-PCR analysis

One ug of total RNA from each cell line and two normal lymph node controls was used to perform qRT-PCR to validate GEP as previously described [11]. qRT-PCR was performed using Applied Biosystems OneStepPlus Real-Time PCR system (Life Technologies) and a cycling protocol with an initial denaturation at 95 °C for 3 min; followed by 40 cycles of 95 °C for 3 s, 62 °C for 20 s, and 72 °C for 15 s; with a final extension at 72 °C for 5 min, followed by a melt curve analysis. All assays were performed in triplicate.

Primers were designed using NCBI Primer-BLAST as previously described [11] for *MYC, KIT, FLT3, PTEN*, and *RPL32* (Table 2). Relative quantification using the comparative Ct method (ΔΔC_T_) was performed as described previously using normal lymph node as the baseline for comparisons. *RPL32* was used as the reference gene based on its stable expression across all samples in GEP analysis and previous identification as a suitable qRT-PCR reference gene for canine lymphoid neoplasia [51].

**Table 2:** Primer sequences and associated cDNA amplicons length used for qRT-PCR analysis

Primer	Sequence	Amplicon length
MYC-F	5′-TCGCCTATTTGGGAAGACAC-3′	141
MYC-R	5′-AAGCTGACGTTGAGAGGCAT-3′	
KIT-F	5′-CGAAGATGTGTGAAGCAGGA-3′	126
KIT-R	5′-GTGTCCGCTACCCTGGAATA-3′	
PTEN-F	5′-ACTTTGAGTTCCCTCAGCCA-3′	141
PTEN-R	5′-AGGTTTCCTCTGGTCCTGGT-3′	
FLT3-F	5′-CAGAGGCAGTGTATGGAGCA-3′	129
FLT3-R	5′-GGCAATTCAGGGAACTGTGT-3′	
RPL32-F	5′-ATGCCCAACATTGGTTATGG-3′	180
RPL32-R	5′-CTCTTTCCACGATGGCTTTG-3′	

## Results

### Immunophenotyping

Flow cytometry data are presented in Table 3. All cell lines expressed CD45 and CD45RA, and 4/5 expressed CD18, which verifies a leukocytic origin. CLBL-1 displayed positive staining for CD1a, CD1c, CD11a, CD11b, CD54, CD79α, CD80, CD86, and MHC-II and no staining for CD3, CD4, CD5, CD8, and Thy-1 indicating a mature B-cell origin, which is further supported by a monoclonal product resulting from PCR analysis of the *IGH* gene. Ema was positive for Thy-1, a T-cell marker. TL-1 cells were negative for most antigens, however weak expression of MHCII was observed, which may indicate a T-cell phenotype as MHCII is expected to have high intensity on B cells and antigen presenting cells, with much weaker expression observed on T cells [52]. TL-1 and Ema both were found to have rearrangement of *TCRG* gene (oligoclonal for TL-1), further supporting a mature T-cell phenotype for both cell lines. UL-1 expressed most antigens including CD34, which indicates an immature precursor phenotype. Positive expression for CD3, CD4, CD5, CD8α, CD8β, CD11d, and Thy-1 all support a T-cell phenotype. UL-1 is also positive for CD14, which is normally a marker for myeloid cells; however, expression is also demonstrated in B and T cell precursors but not mature cells [52], further supporting an immature phenotype. Additionally, PCR of *TCRG* indicated a monoclonal product further supporting T-cell phenotype. 3132 is most likely of dendritc/histiocytic origin based on the positive expression of CD1a and CD11c, which is characteristic of histiocytic diseases [53] and a combination not found in other cell lines of lymphoid origin in this or previous studies [10, 11]. Additionally, strong intensity for MHC-II further supports dendritic cell origin. However, a T-cell origin cannot be completely ruled out based on expression of CD4, CD8α, and a monoclonal product resulting from PCR of *TCRG*.

**Table 3:** Phenotypic characteristics of canine LL cell lines based on flow cytometry analysis

Cell line	CLBL-1	Ema	TL-1	UL-1	3132
Antigen					
CD1a	++	–	–	+	+
CD1c	++	–	–	+	++
CD3	–	–	–	+	–
CD4	–	–	–	+	+
CD5	–	–	–	+	–
CD8α	–	–	–	++	++
CD8β	–	–	–	+	–
CD11a	+	+	–	–	+
CD11b	+	–	–	–	++
CD11c	–	–	–	–	++
CD11d	–	–	–	+	–
CD14	–	–	–	+	+
CD18	++	++	–	++	++
CD21	–	–	–	+	–
CD34	–	–	–	+	–
CD45	++	++	++	+	++
CD45RA	++	+	+	+	+
CD49α	++	++	–	++	++
CD54	++	–	–	+	++
CD79α/β	++	–	–	–	–
CD80	+	++	++	+	++
CD86	+	–	–	+	++
DM5	–	–	–	+	–
MHC-II	++	–	+	+	++
TCRαβ	–	–	–	+	–
TCRγδ	–	–	–	+	+
Thy-1 (CD90)	–	++	–	+	–

Strong expression (++), intermediate expression (+), or no expression (−) of each antigen is indicated

### Karyotype architecture

**Fig. 1:**
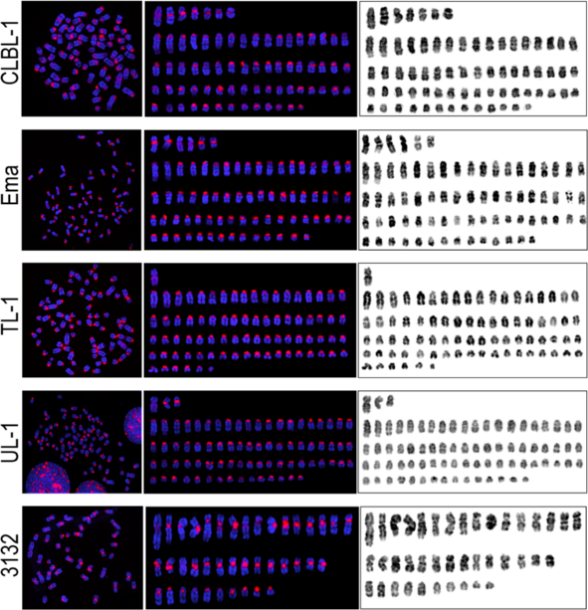
Karyotypic organization of each canine LL cell line. Centromeric regions were visualized through the use of two BAC clones, and used to orient the chromosomes. Images of DAPI stained metaphase spreads (left panel) were used to prepare rudimentary karyotypes (middle and right panels). Chromosomes were arranged by descending size of bi-armed chromosomes followed by single-armed chromosomes

Chromosome enumeration and centromere localization via FISH of metaphase chromosomes was used to assess the gross karyotypic architecture of each cell line. The normal dog karyotype includes 38 pairs of acrocentric autosomes, a large sub-metacentric X chromosome and a small metacentric Y chromosome [54]. Enumeration of chromosomes from 30 metaphase spreads of each cell line indicated varying levels of aneuploidy (Fig. 1). CLBL-1, Ema, and 3132 were all hypodiploid, while TL-1 and UL-1 contained normal modal chromosome counts (Table 4).

**Table 4:** Chromosome enumeration of five canine LL cell lines based on evaluation of 30 metaphase spreads

Cell line	Ploidy	Modal chromosome number	Chromosome range	Bi-armed chromosomes
CLBL-1	Hypodiploid	70	66–72	4–7 per cell
Ema	Hypodiploid	73	70–76	5–7 per cell
TL-1	Normal	78	76–82	1 per cell
UL-1	Normal	78	76–80	1–3 per cell
3132	Hypodiploid	40	37–42	25–34 per cell

### Genome wide copy number imbalance

**Fig. 2:**
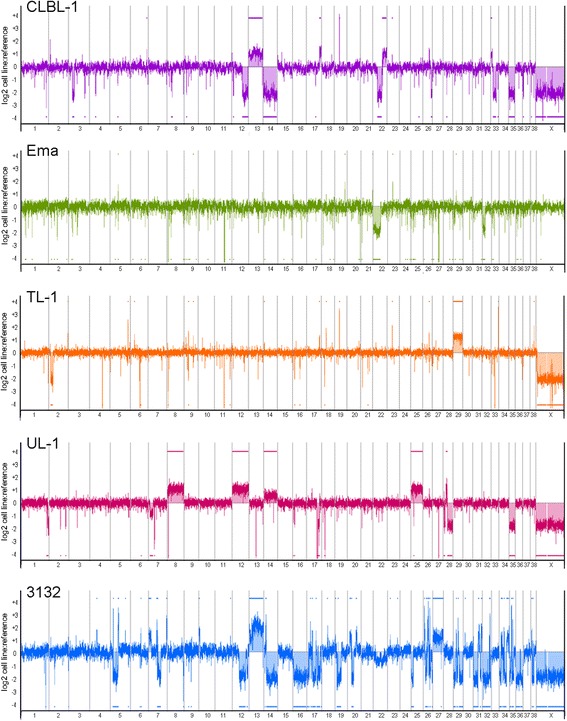
oaCGH profiles of each of the five canine LL cell lines. Each oaCGH profile includes the chromosomes (1–38,X) on the x-axis and log2 cell line:reference ratio on the y-axis with copy number gains and losses indicated by the horizontal bars above and below the midline, respectively

**Fig. 3:**
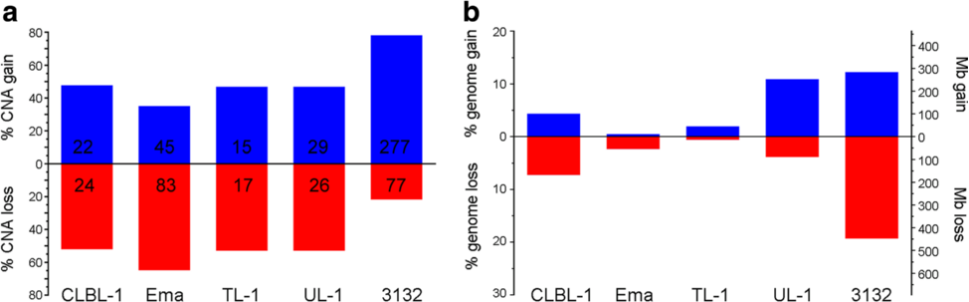
Genomic imbalances identified in the five canine LL cell lines. **a** Percentage of CNAs detected as gain or loss via oaCGH analysis, with the total number indicated on each column. **b** Genomic imbalances in each cell line expressed as percent genome changed and the total number of megabases (Mb) within regions of copy number change. Copy number changes on the X chromosome were omitted from these analysis as the reference was sex mismatched in 4/5 cell lines

**Fig. 4:**
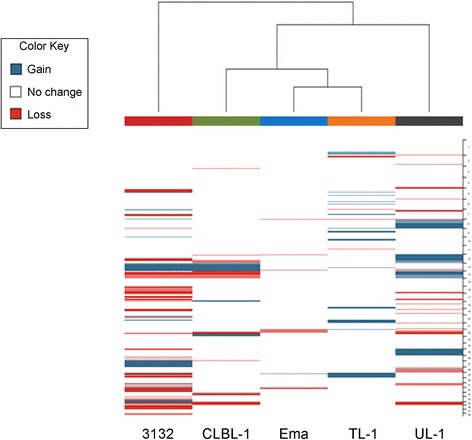
Hierarchical clustering of canine LL cell lines based on genome wide copy number status. Dichotomous data was clustered using Euclidian distance and Ward’s method. Columns represent the individual cell lines and rows represent individual regions along the genome. Blue indicates a region of gain and red indicates a region of loss

oaCGH analysis revealed varying levels of genomic imbalance in each of the cell lines with detected CNAs ranging in size from 19.5 Mb to entire chromosomes (Fig. 2). Large differences in the number of aberrations (Fig. 3a) and the percent of the genome impacted by regions of imbalance (Fig. 3b) were also noted. CLBL-1, TL-1, and UL-1 have a similar and even distribution of the number of aberrant gains and losses; whereas Ema was skewed towards an increase in the number of losses, and 3132 was skewed towards regions of copy number gain. When the cumulative size of CNAs and the percent of the genome impacted by imbalance were assessed, Ema and TL-1 were minimally changed which is reiterated from the oaCGH profiles in which both cell lines had less visually obvious CNAs. CLBL-1 and UL-1 had similar total percent of the genome changed, but CLBL-1 had a greater percent of the genome with imbalances of loss and UL-1 had a greater percent of the genome gained. 3132 had the greatest percent of the genome changed which was expected given the number of aberrations detected and the extensive genomic complexity visualized in the oaCGH profile. Cancer-associated genes in regions of imbalance were identified and are detailed in Additional file 1: Table S1. Cell lines were clustered to determine their relatedness to each other based on genome-wide copy number assessment (Fig. 4). 3132 branched away from the other four, and Ema and TL-1, the two mature T-cell neoplasms based on immunophenotyping, were the most closely related.

### FISH analysis

**Fig. 5:**
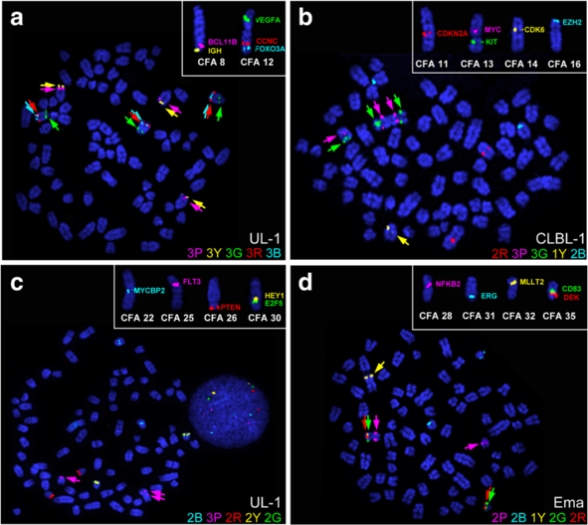
FISH analysis of 20 cancer-related genes in each canine LL cell line verifies and enumerates oaCGH findings and identifies structural aberrations. The 20 genes were divided into four panels of five genes each for multicolor FISH (**a**-**d**). In each panel, individual chromosomes from clinical healthy control dogs are included in the inset to show normal probe placement. For each gene panel, a representative metaphase spread showing copy number or structural aberrations (arrows) is included. Cell line and copy number of each probe are denoted in each panel

FISH analysis was completed for 20 genes relevant to lymphoid neoplasia to verify oaCGH data, enumerate the level of imbalance at each locus, and investigate any structural aberrations associated with the selected genes of interest. The 20 genes were divided into four panels of five for multicolor FISH analysis. Each of the four FISH panels is identified in the insets of Fig. 5 with control chromosomes from clinically healthy dogs indicating the appropriate localization of each BAC clone. A representative metaphase spread with CNAs or structural changes in one of the cell lines is shown in each panel.

Structural changes were identified in each cell line. CLBL-1 contains a derivative 13 chromosome that appears to include two copies of *Canis familiaris* 13 (CFA 13) joined by the centromere resulting in the formation of a metacentric chromosome (Fig. 5b), in addition to a grossly normal copy of CFA 13 based on the location of the 2 probes on CFA 13. A similar abnormality occurred in UL-1 with chromosome 25, as is evidenced by 2 copies of *FLT3* forming a metacentric chromosome in addition to an acrocentric chromosome with *FLT3* placement in the expected location on CFA 25 (Fig. 5c). Both Ema and *CLBL-1* contained a derivative metacentric chromosome resulting from the apparent fusion of CFA 28 and CFA 35 (Fig. 5d). Ema contained additional copies of CFA 28 and CFA 35, while *CLBL-1* contained another CFA 28 and a heterozygous loss of CFA 35. Numerous structural aberrations occurred in 3132, and appeared to be random from cell to cell. Further detail of these structural changes was difficult to ascertain given the heterogeneity observed in the metaphase spreads.

**Fig. 6:**
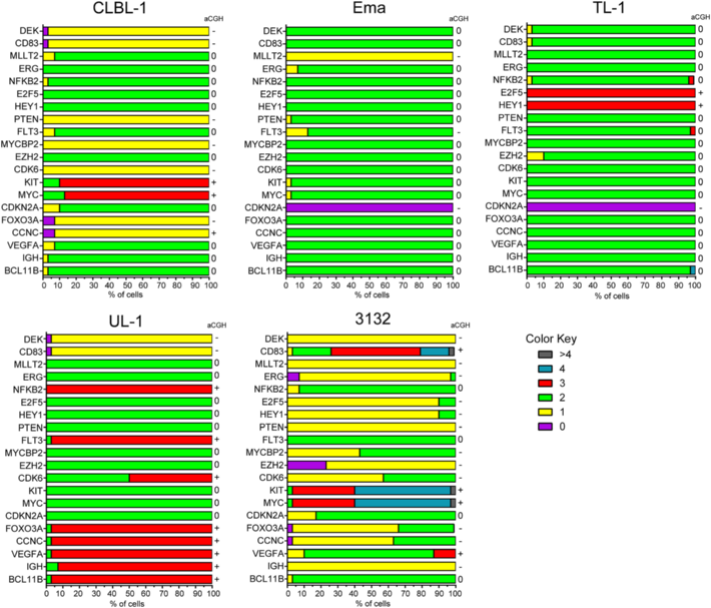
Summary of CNAs identified in FISH analysis of 20 cancer-related genes in each canine LL cell line. Each chart shows the distribution of copy number for each probe, based on the analysis of 50 cells from each cell line. Data are stacked to represent the percentage of cells displaying copy numbers 0 to >4 as indicated by the color key. To the right of each chart, dichotomized oaCGH data as gain (+), loss (−) or no change (0) is notated

A summary of the frequency of CNA at each of the 20 investigated loci is presented in Fig. 6. Ema and TL-1 showed grossly normal copy number changes, as was seen in the oaCGH; however, both were found to have a homozygous deletion of *CDKN2A*. Ema also had a heterozygous loss of *MLLT2* in 100 % of cells and a heterozygous loss of *FLT3* in 15 % of cells which was detected via oaCGH. Additionally, TL-1 had three copies of *E2F5* and *HEY1*, both located on CFA 30, in 100 % of cells. UL-1 and CLBL-1 showed copy number changes in nine and 10 of the loci evaluated, respectively. CLBL-1 exhibited heterozygous losses of *CCNC, FOXO3A, CDK6, MYCBP2, PTEN, CD83,* and *DEK*, and a copy number of three for *MYC* and *KIT* (both located on CFA 13) in the majority of cells (>87 %). UL-1 had more genes gained, with three copies of *BCL11B, IGH, VEGFA, CCNC, FOXO3A, FLT3*, and *NFKB2* in the vast majority (>93 %) of cells. Additionally, 50 % of cells gained an extra copy of *CDK6*. FISH analysis of 3132 showed copy number changes at 19/20 loci, many of which were strikingly heterogeneous. *MYC, KIT*, and *CD83* each have proportions of cells with normal copy number and gains of 3, 4, and >4 copies. Losses of *ERG* and *EZH2* were identified in all or nearly all 3132 cells, with proportions (10–23 %) of cells displaying homozygous deletion.

### Gene expression analysis

**Fig. 7:**
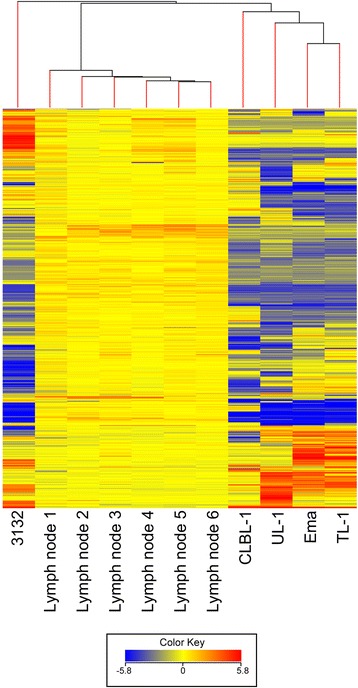
Unsupervised hierarchical clustering of gene expression data from five canine LL cell lines and six non-neoplastic control lymph nodes. Data were filtered to remove transcripts displaying limited variability (standard deviation <2) resulting in 1153 probe sets used for clustering analysis using Euclidian distance and Ward’s method

GEP data were filtered to remove probe sets with limited variation (standard deviation <2) across the five cell lines and six non-neoplastic lymph nodes resulting in 1153 probe sets used for subsequent analyses. Unsupervised clustering resulted in immediate branching of 3132 from the other 10 samples. The remaining samples were then separated into two discrete groups including the four cell lines, and the six control lymph nodes (Fig. 7). Normal lymph nodes unsurprisingly display greater transcription conservation across biological replicates compared with the cell lines as indicated by their shorter connecting branches. The cell lines displaying the greatest transcriptional similarity were Ema and TL-1, the two mature T-cell lines, as reiterated from the oaCGH clustering analysis. UL-1, most likely an immature T-cell phenotype, is also more similar to Ema and TL-1 than CLBL-1, a B-cell line.

Fold change analysis was also completed by comparing each cell line with the mean expression of the non-neoplastic lymph nodes. Fold changes of known cancer related genes and the transcripts with the 50 largest absolute fold changes (up or down) in each cell line are listed in Additional file 1: Tables S2 and S3, respectively, as genes that may have functional relevance. Additionally, genes with a fold change >5 were further assessed in functional gene-annotation enrichment analysis. Due to limited functional annotation of canine genes, official gene symbols were used for enrichment analysis based on human gene annotations. Up to the top ten enriched GO biological processes and KEGG pathways are listed in Additional file 1: Tables S4 and S5, respectively. All five cell lines exhibited upregulation of genes associated with the GO biological processes of cell proliferation and division, and three of five exhibited upregulation of genes involved in metabolic processes (e.g. sterol biosynthetic process, glucose metabolic process, cholesterol metabolic process). All five cell lines also exhibited downregulation of genes involved in immune response and leukocyte activation. When the KEGG pathways were assessed, four of the cell lines exhibited upregulation of metabolic and biosynthesis pathways (e.g. steroid biosynthesis, pyruvate metabolism) as similarly identified in the GO term analysis. TL-1 was also found to exhibit upregulation of genes in both the ERBB and Jak-STAT signaling pathways; while genes involved in the MAPK signaling pathways and the NOD-like and Toll-like receptor signaling pathways were upregulated in 3132. All cell lines exhibited downregulation of cell adhesion molecules.

**Fig. 8:**
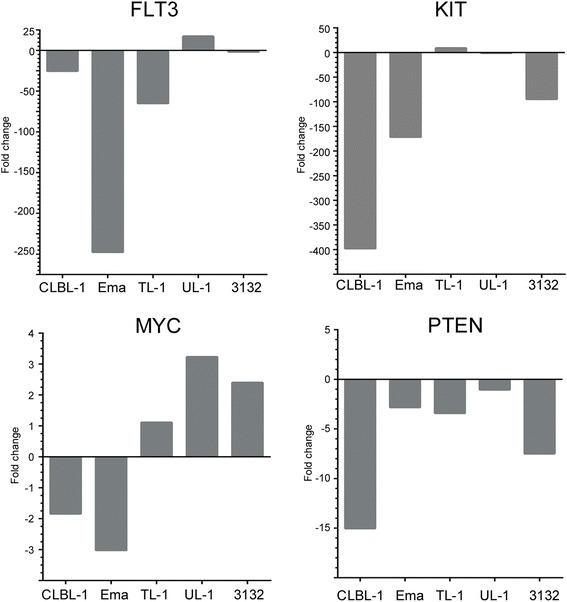
qRT-PCR analysis of gene expression in five canine LL cell lines. Transcriptional levels of *FLT3*, *PTEN*, *MYC*, and *KIT* were assessed to verify microarray changes and further analyze the relationship between copy number change and expression change. *RPL32* was used as a reference gene to normalize expression levels between samples, and fold changes were calculated relative to the average expression in two non-neoplastic lymph nodes

qRT-PCR was performed to verify GEP findings and analyze the relationship between copy number change and expression change in four well known cancer associated genes including *MYC, KIT, FLT3*, and *PTEN* (Fig. 8). Homozygous loss of *PTEN* was observed in CLBL-1 and 3132, which both show the greatest decreases in *PTEN* expression. Three copies of *FLT3* were present in UL-1, and this gene was upregulated 17 fold (and was the only cell line to show an upregulation). Fold changes in *MYC* and *KIT* are more variable across the cell lines and have minimal correlation with copy number changes observed. CLBL-1 and Ema had slight downregulation of *MYC*, while the other three showed modest upregulation. *KIT* was largely downregulated in CLBL-1, Ema, and 3132 (two of which had copy number gains), and upregulated 8-fold in TL-1 in which the loci was copy number neutral.

### Genome-wide CNA comparison with primary tumors

**Fig. 9:**
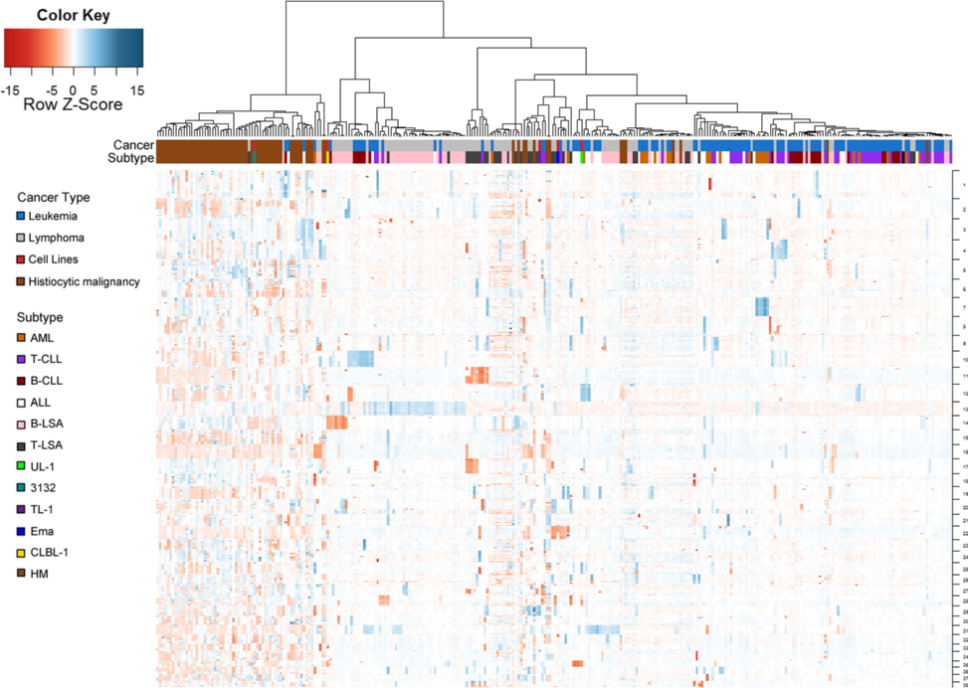
Hierarchical clustering of canine LL cell lines with 299 primary canine round cell tumors including 123 leukemias, 106 lymphomas, and 70 histiocytic malignancies based on genome wide copy number status. Data consisted of segmented values that were scaled and clustered using Euclidian distance and Ward’s method. Columns represent individual patients and rows represent individual markers along the genome. Blue indicates a region of gain and red indicates a region of loss. Cancer type and Subtype metadata is annotated for each column. Abbreviations are as follows: Acute myeloid leukemia (AML), T-cell chronic lymphocytic leukemia (T-CLL), B-cell chronic lymphocytic leukemia (B-CLL), acute lymphoblastic leukemia (ALL), B-cell lymphoma (B-LSA), T-cell lymphoma (T-LSA), and histiocytic malignancy (HM)

Finally, we compared the five cell lines with genome wide copy number data generated in our laboratory from 299 primary round cell tumors via clustering analysis to determine whether the cell lines represented primary tumors, and, if so, what subtype each most closely resembled (Fig. 9). All cell lines were found to cluster within the primary tumors, indicating they are more closely related to primary tumors than other cell lines. 3132 segregated within a large cluster composed of primarily histiocytic malignancies. CLBL-1 branches from a cluster of mature B-cell neoplasms consisting primarily of B-cell lymphomas with a smaller subset of B-cell chronic lymphocytic leukemias. TL-1 and Ema both clustered in a somewhat heterogeneous grouping comprised chiefly of T-cell lymphomas, and both these cell lines were most closely related to a T-cell lymphoma. UL-1 grouped in a cluster composed of acute leukemias, most of which were lymphoid in origin.

## Discussion

We present a detailed characterization of five canine LL cell lines using a genome-wide molecular approach including oaCGH and GEP analysis. The data presented here, combined with previously published canine LL cell line characterizations [11], provides the opportunity to more appropriately select canine LL cell lines for preclinical in vitro studies. Overall, our data suggests that CLBL-1 represents a mature B-cell lymphoma as previously reported [22], and Ema and TL-1 both represent T-cell lymphomas as previously reported [23]. In contrast, UL-1 should be reclassified as T-cell acute lymphoblastic leukemia (instead of a T-cell lymphoma) and 3132 should be reclassified as a histiocytic sarcoma (instead of a B-cell lymphoma).

CLBL-1 immunophenotyping in this study agreed with the immunophenotyping published when the cell line was initially established. Positive expression of CD11a, CD79α, CD45, CD45RA, and MHCII and no expression of CD3, CD4, CD5, CD8, CD11d, CD14, CD21, CD34, and CD56 classifies it as a B-cell lymphoma. Additionally, B-cell PARR revealed a monoclonal rearrangement, which we also found [22]. CLBL-1 was the only cell line in this dataset that had been previously karyotyped [55]. It was found to be hypodiploid with a modal chromosome number of 70–71, which matches our findings. There was only one biarmed chromosome mentioned, derivative chromosome 13, which we also identified via FISH analysis. Since centromere visualization was not previously completed, and a non-standard canine chromosome nomenclature was used, correlation of further previously identified chromosome aberrations with our findings via DAPI banding, FISH, and oaCGH was not possible. Numerous CNAs identified in CLBL-1 via oaCGH are shared with primary canine B-cell lymphomas (B-LSA). Gain of CFA 13 has been identified in 25 % of B-LSA. Loss of CFA14 and loss of the proximal region of CFA 3 have also been found to occur in ~10 % of B-LSA [8]. Additionally, CLBL-1 clustered with primary mature B-cell lymphoid neoplasia when clustered with 299 primary canine round cell tumors, further supporting the classification of CLBL-1 as a B-cell lymphoma.

Ema immunophenotyping, at the time of establishment, was positive for CD3, CD45, CD45RA, and Thy-1. In our assessment, Ema was also positive (intermediate signal) for CD11a and CD18 and negative for CD3. Similarly, TL-1 was originally positive for CD3, CD18, CD45, CD45RA, and MHC11 while our immunophenotyping indicated no expression of CD3 and CD18. The slight differences in the immunophenotyping data may be due to factors that can influence flow cytometry data including, the amount of antibody used, the call threshold of positive versus negative, instrumentation, and other reagents. Alternatively, it’s possible that the loss or gain of antigens on the cell surface occurred while in culture. Both Ema and TL-1 were previously T-cell PARR positive, which is consistent with our PARR data. Overall, the changes observed in immunophenotype did not impact the overall interpretation of the classification of both these cell lines as mature T-cell lymphoid neoplasia.

Both Ema and TL-1 were found to have a copy number loss in the region containing *CDKN2A* in oaCGH analysis, which was further confirmed to be a homozygous loss of the gene loci in FISH analysis. *CDKN2A* loss occurs in >55 % of T-cell lymphomas (T-LSA) and <2 % of other subtypes of canine leukemia and lymphoma [8, 34]. Ema was also found to have a loss of the proximal half of CFA 22, which occurs in 20 % of T-LSA, and TL-1 had a gain of CFA 29 previously identified in 40 % of T-LSA [8]. Ema and TL-1 were also the cell lines most closely related in clustering analyses using both the oaCGH and GEP data, and both segregated with primary T-LSA when clustered with primary canine round cell tumors.

UL-1 immunophenotyping at the time of establishment was positive for CD8α, CD18, CD45, CD45RA. We found additional positive expression of CD3, CD4, CD5, CD14, CD21, CD34, Thy-1, and MHC-II. All were detected at an intermediate level therefore, as discussed earlier, differences in methods and data analysis could have resulted in previous negative findings. UL-1 was also T-cell PARR positive which agrees with our PARR findings. UL-1 was previously classified as a T cell lymphoma; however, based on expression of CD34, a surface glycoprotein expressed on hematopoietic stem cells, we suggest it is more representative of a T-cell acute leukemia (T-cell ALL). In veterinary medicine, CD34 expression can be used to suggest an acute leukemia since it is less commonly expressed in malignancies of more mature cells, such as lymphomas and chronic lymphocytic leukemias [26, 56]. Several regions of CNA are also shared with primary canine ALLs including the loss of the distal end of CFA 1 which occurs in 20 % of ALLs, gain of CFA 12 and 25 which occurs in 15 and 10 % of ALLs, respectively, and loss of CFA 35 which occurs in 15 % of ALLs. Of the CNAs identified in UL-1, more were in common with ALLs than other subtypes. UL-1 also segregated with a group of ALLs when clustered with primary round cell tumors. Together, these findings support the classification of UL-1 as a T-cell ALL.

3132 immunophenotyping in our laboratory, in conjunction with the original description of the cells (cellular pleomorphism, multinucleated giant cells, numerous mitotic figures, extreme variations in nuclear:cytoplasm ratio) suggests this cell line is representative of a disseminated histiocytic sarcoma (HS). No immunophentypic or genomic analysis of this cell line has been previously reported, although it was later described as a B-cell lymphoma based on detectable surface immunoglobulins [24], and it has since been cited in the literature as a B-cell lymphoma [21]. Our immunophenotyping revealed expression of CD1, CD11c, CD18, and MHCII which is the characteristic immunophenotype of canine HS [53, 57]. Since the cells also exhibit low and intermediate expression of CD4 and TCRδγ, respectively, we could not completely rule out a T-cell lymphoma with aberrant expression of CD11c [52, 53], although subsequent genomic results further supported HS. 3132 cells were also T-cell PARR positive, although rearrangements of both T-cell receptor and immunoglobulin genes have been previously identified in human histiocytic sarcomas [1, 58] and in canine histiocytomas [53]. Finally, our karyotypic, CNA, and GEP data also suggests that 3132 cells represent a histiocytic sarcoma. Metaphase spreads from primary cases contain highly variable chromosome numbers with a range of 42–53 and an abundance of aberrant bi-armed chromosomes which is comparable to karyotypic findings of 3132. oaCGH of 3132 cells was indicative of high genomic instability based on the number of CNAs identified, the percentage of the genome involved in regions of CNA, and the observation that several chromosomes include numerous of gains and losses across the chromosome, all of which have been found in primary HS [59]. Several specific CNAs are also shared with primary HS, including loss of CFA 16 and loss of CFA 31. Loss of CFA 12, 14, and 36 and gain of CFA 13 are also conserved with CNAs in primary canine HS [59]. When examining cluster analysis of GEP data with primary canine round cell tumors, 3132 segregated with a large group of histiocytic malignancies.

Dysregulation of several genes previously associated with canine diffuse large B-cell lymphoma in the NF-kB signaling and B-cell receptor signaling pathways shared conserved expression in CLBL-1 including the following: *KRAS, NRAS, PIK3R5, PLCG2, TGFBR2, TNFAIP3, TRADD, BCL2A1, CAMK2D, NFATC2* [48], *BUB1B, PRKCD, CD83, CXCL13, CD36, IL8, IL2, CD40LG, LCK, LTBR,* and *TNFSF11* [21]. Similarities were also noted between CLBL-1 and other targeted B-cell lymphoma gene expression studies, including *KIT* (almost 400-fold based on qRT-PCR) [60] and *ZAP70* downregulation (decreased 10 fold) [61].

There are less reported data regarding gene expression changes in other canine hematopoietic malignancies. *KIT* expression is decreased in canine T-cell LSA [62], and was decreased three fold in the TL-1 cell line. *SYK* expression is downregulated in canine T cell malignancies [61] and was downregulated 12–14 fold in all three T-cell cell lines (Ema, TL-1, and UL-1). *MMP9* and *TIMP1* expression is significantly upregulated in canine T-cell lymphomas [13], and was upregulated in both Ema and TL-1 cells. UL-1 was the only cell line to upregulate *FLT3*, and increased *FLT3* expression has been previously identified in canine a subset of acute lymphocytic leukemias and the GL-1 cell line [9]. Upregulation of *VEGFA* was also found in UL-1 and has been previously reported in canine ALL [62]. Finally, upregulation of *GTSF1, LUM*, and *PYPH* and downregulation of *CLEC12A* and *CD9* in primary canine histiocytic sarcomas [63, 64] were found to be dysregulated similarly in 3132 cells.

Cross contamination of cell lines is a common issue in the scientific community with ~19 % of human LL cell lines being cross contaminated [6]. This problem is further evidenced by the identification of human cells in five recently characterized canine B-cell lymphoma cell lines [65]. Our cytogenetic analysis of chromosomal architecture, in conjunction with oaCGH and FISH analysis, proves none of the examined cell lines are cross contaminated with other canine or human cells.

## Conclusion

In summary, we present the comprehensive immunophenotypic and genomic characterization of 5 canine LL cell lines that confirms their cell of origin in 3 cell lines (CLBL-1, Ema, and TL-1) and refutes their cell of origin in 2 cell lines (UL-1 and 3132). These data provide valuable information that can be used to select cell lines for preclinical in vitro studies based on the presence or absence of particular immunophenotypic and/or genomic characteristics of interest, which will enhance their clinical predictive value. The generation of integrated molecular profiling of cell lines and comparison with primary tumors will allow further exploration into their biology and clinical utility in veterinary medicine and contribute to comparative and translational studies of hematopoietic malignancies in dogs and humans.

## Additional file

Additional file 1: Table S1. Known cancer-associated genes located in regions of genomic imbalance in each of the five cell lines. **Table S2.** Summary of fold changes of cancer-associated genes for each cell line when normalized expression level was compared with mean normalized expression levels of non-neoplastic lymph nodes. **Table S3.** List of 50 transcripts from each cell line with the largest absolute fold changes when normalized expression level was compared with mean normalized expression levels of non-neoplastic lymph nodes. **Table S4.** Gene ontology (GO) biological processes enriched for genes upregulated or downregulatued by >5 fold in each cell line when compared with non-neoplastic lymph nodes. The top ten terms associated with up and down regulated genes are listed. **Table S5.** KEGG pathways associatated with genes upregulated or downregulated by >5 fold in each cell line when compared with non-neoplastic lymph node. Up to the top ten pathways associated with up or down regulated genes are listed. (XLSX 113 kb)

## Abbreviations

CNA: Copy number analysis
FISH: Fluorescent in situ hybridization
GEP: Gene expression profiling
LL: Leukemia/lymphoma
oaCGH: Oligonucleotide array comparative genomic hybridization
PARR: Polymerase chain reaction for antigen receptor rearrangement
